# Detection of human papillomavirus infection in oral mucosal diseases: A single-center study

**DOI:** 10.1016/j.jfscie.2024.100031

**Published:** 2024-04-15

**Authors:** Paolo Junior Fantozzi, Umberto Romeo, Gianluca Tenore, Gaspare Palaia, Chiara Ciolfi, Alessandra Pierangeli, Cira Rosaria Tiziana Di Gioia, Alessandro Villa

**Affiliations:** a Department of Oral and Maxillofacial Sciences, Sapienza University of Rome, Rome, Italy; b Laboratory of Microbiology and Virology, Department of Molecular Medicine, Sapienza University of Rome, Rome, Italy; c Department of Radiological, Oncological, and Anatomic Pathological Sciences, Sapienza University of Rome, Rome, Italy; d Department of Orofacial Sciences, University of California San Francisco, San Francisco, CA; e Oral Medicine, Oral Oncology and Dentistry, Miami Cancer Institute, Baptist Health South Florida, The Herbert Wertheim College of Medicine, Florida International University, Miami, FL

**Keywords:** Oral medicine, oral HPV, Human papillomavirus, oral leukoplakia, oral cancer, oral infections

## Abstract

**Background:**

Human papillomavirus (HPV) infection accounts for more than 70% of oropharyngeal cancers but only a small proportion of oral cavity cancers. This study aimed to investigate the presence of HPV DNA in oral diseases to understand better the possible correlation between oral lesions and HPV infections.

**Methods:**

This was a cross-sectional study of 99 adult patients seen for the evaluation of oral diseases. All patients received an oral biopsy and histopathologic examination and a brush biopsy for HPV-DNA detection and genotyping by real-time polymerase chain reaction. Immunohistochemistry was used to assess p16INK4a expression.

**Results:**

HPV was identified in 15 of 99 (15.2%) patients (males, 66.6%). Patients with oral leukoplakia (OL) (46.6%), followed by patients with oral lichen planus (OLP) (33.3%) had the highest rate of HPV infection, with a predilection for the buccal mucosa (17.5%). Most patients with high-risk HPV infections had OLP (4/10, 40.0%), whereas most of the patients with low-risk HPV infections had nonreactive epithelial hyperkeratosis (3/6, 50.0%). Among all benign lesions, 19.0% were positive for any HPV infection. One patient with OL showing mild epithelial dysplasia had a positive p16INK4a expression.

**Conclusions:**

The highest rate of HPV infection was in male patients, patients with OL and OLP, and conditions affecting the buccal mucosa. Larger studies are needed to elucidate the role of HPV in the development of these conditions.

## Graphical abstract

**Figure:**
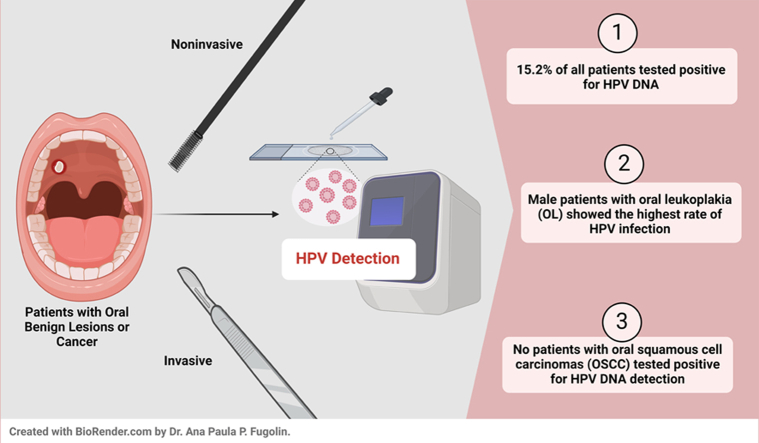

**Why Is This Important?**

Human papillomavirus (HPV) is a common sexually transmitted infection with a global prevalence of around 12% and with more than 80% of the global population being infected at some point in their lifetimes. Persistent oral and oropharyngeal HPV infection of high-risk types (mainly HPV-16 and HPV-18) is associated with more than 70% of oropharyngeal cancers and a small percentage (3%-5%) of oral squamous cell carcinomas (OSCC). OSCC is the most common type of oral cancer and often arises from oral potentially malignant disorders through a series of genetic alterations; given the heightened numbers of HPV-positive oropharyngeal squamous cell carcinomas but a small number of HPV-positive OSCC, this study aimed to investigate the presence of HPV in patients with oral lesions using both invasive and noninvasive detection methods. In particular, the prevalence of HPV infection in benign, potentially malignant, and malignant conditions of the oral cavity was assessed.

## Introduction

Human papillomavirus (HPV) is a common sexually transmitted infection with a global prevalence of 11.7%. In addition, there are 387 million new cases in Europe each year, and more than 80% of the global population has been infected at some point in their lifetimes.^1^^,^^2^ HPV belongs to a family of small DNA viruses with a specific tropism for epithelial tissues affecting cutaneous and mucosal areas through direct contact with known risk factors that include oral sex, young age, sexual intercourse, number of lifetime sexual partners, tobacco and marijuana smoking, and immunosuppression (eg, history of HIV).^3^^,^^4^ Although sexual intercourse is the most documented HPV route of transmission, there have been cases reporting nonsexual driven HPV infections, mainly secondary to fomites or nonsexual skin-to-skin or skin-to-mucosa contacts (fingers-to-mouth or fingers-to-skin contact).^5^ Although most HPV infections are asymptomatic and resolve spontaneously within 1 or 2 years, depending on the site considered, persistent HPV infection may lead to benign or malignant pathology.^6^

HPV genotypes are classified as low risk (genotypes: 6, 11, 40, 42, 43, 44, 53, 54, 61, 72, 73, 81) that are responsible for most genital and oral warts, and high risk (oncogenic genotypes: 16, 18, 31, 33, 35, 39, 45, 51, 52, 56, 58, 59, 68) that are responsible for precancerous lesions, oropharyngeal cancer (OPC), cervical cancer, and malignancies of the vulva, vagina, penis, or anus.^7^ Approximately 5% of all cancers worldwide are caused by HPV, of which HPV-16 and HPV-18 account for most of the HPV-related cancer burden.^8^ Most cancers attributed to HPV are preventable, including OPCs.^9^^,^^10^ The 9-valent HPV vaccine is approved in several countries for the prevention of vaginal, vulvar, anal, oropharyngeal, and other head and neck cancers.^11^ Although the vaccine is one of the most effective cancer prevention tools available, many adolescents and their parents remain unaware of the role of the HPV vaccination in OPC.^12^

Oral HPV infection among healthy adults aged 18 through 69 years has an overall global prevalence of 7.7%, with increased numbers in men (9.3%), patients from South America (12.4%), and people at higher risk of HPV infections (ie, patients with immunosuppression and patients having multiple partners) (12.5%).^13^^,^^14^

Most oral conditions associated with HPV infection are benign and include squamous papilloma, condyloma acuminatum, verruca vulgaris, and Heck disease (or focal epithelial hyperplasia); the latter condition manifests as multifocal sessile nodules of the oral mucosa, particularly seen in young people in Latin American countries.^15^ Pyogenic granuloma, a benign condition of vascular and inflammatory origin, has also been associated with HPV type 2 infection.^16^

Persistent oral and oropharyngeal HPV infection of oncogenic genotypes (mainly HPV-16 and HPV-18) is associated with most OPCs, but only a small percentage of oral squamous cell carcinomas (OSCC), which for most cases are caused by non-HPV risk factors (ie, chronic alcohol abuse and tobacco consumption), and often arise from oral potentially malignant disorders (OPMD) through a stepwise series of genetic alterations.^17^ In Western countries, oral leukoplakia (OL) represents the most common OPMD and has global prevalence rates that range from 1.5% through 2.6% with no sex predilection.^18^ Clinically, OL can be subclassified as localized leukoplakia and proliferative leukoplakia (also called proliferative verrucous leukoplakia), each of which has distinct risk factors and clinical behavior^19^^,^^20^ Localized leukoplakia is usually a solitary lesion, strongly associated with tobacco use, and more common in men, and it has a malignant transformation rate ranging from 1% through 41%.^21^ Proliferative leukoplakia typically manifests as a multifocal condition, shows a weaker association with tobacco use, is more frequently observed in women, and has a malignant transformation rate ranging from 60% through 90%.^22^^,^^23^ Several studies have explored the association between HPV infection and OLs. A 2021 meta-analysis showed that among 1,157 patients with OSCCs, the rates of high-risk HPV infection (HPV-16) were 21.5% in patients with tongue squamous cell carcinoma and 15.7% in patients with floor of the mouth SCC.^24^ Another large systematic review and meta-analysis evaluated if persistent high-risk HPV infection could play a role in the development of OPMDs and contribute to the presence of oral epithelial dysplasia.^25^ Among the more than 2,500 OPMDs, 22.5% had detectable HPV DNA. Detection varied both geographically, with South America showing the highest rate (46.8%) and by OPMDs subtypes, with submucous fibrosis showing the highest rate of 28.6%, followed by oral proliferative leukoplakia with 24.7%.^25^

Although the relationship between HPV infection and OPC is well established, the role of HPV infection in the development of OPMDs and OSCC is poorly understood, with only 3% through 5% of cancers of the oral cavity caused by persistent high-risk HPV infection despite the anatomic proximity to the oropharyngeal area.^26^^,^^27^ As such, the objective of this study was to investigate the presence of HPV in patients with oral lesions using both invasive and noninvasive detection methods.

## Methods

### Study design

This was a cross-sectional cohort study of adult patients (≥ 18 years) who were referred to the Unit of Oral Medicine, Department of Oral and Maxillofacial Sciences, Sapienza University of Rome in Italy from March 2016 through September 2017 for the evaluation and management of oral lesions. All participants read and signed a written informed consent. This study included patients with a clinical diagnosis of oral benign lesions (ie, oral fibroma, oral pyogenic granuloma, squamous papilloma), OPMDs, and OSCC. Exclusion criteria included patients living with HIV, patients with a known history of immunosuppression, patients with malignancies of nonepithelial origin, and those with a history of OPC.^28^ Demographic data, social history, information on tobacco and alcohol consumption, and patients’ type and anatomic site of the oral condition were recorded and entered into a deidentified spreadsheet (Excel, Microsoft Corporation). The study was conducted in accordance with the principles of the Declaration of Helsinki^29^ and Good Clinical Practice guidelines^30^ and was approved by the University Hospital institutional review board of the Sapienza University of Rome (IRB N.000873).

### HPV sampling and detection

#### Cytobrush Biopsy

All patients underwent a thorough extraoral and intraoral examination performed by an oral medicine expert. During the first visit, a brush biopsy was obtained using a Cytobrush (GPS Medical), which was applied with a gentle rotating pressure on the lesions to collect a large number of transepithelial cells. The brushing samples were then suspended in 1 mL phosphate-buffered saline and subsequently sent to the virology laboratory for analysis. HPV status was then assessed at the Laboratory of Microbiology and Virology, Department of Molecular Medicine, across the 3 groups (oral benign lesions vs OPMD vs OSCC) using the genotype-specific, real-time polymerase chain reaction (rtPCR) protocol described by Pierangeli et al.^31^

#### Mucosal Biopsy

Alongside the exfoliative cytology, all patients received an incisional or excisional biopsy under local anesthesia to evaluate the histopathologic diagnosis,^32^ the HPV status and nuclear and cytoplasmic reactivity of p16INKa expression via immunohistochemistry (IHC) which was considered positive for valid results when detected at more than 75%.^33^ Criteria for incisional or excisional biopsy were determined depending on the clinical manifestation of the oral lesions (benign vs OPMD vs OSCC), size (< 1 cm or > 1 cm in largest diameter) as well as the location of the lesion for those patients who had already received a histopathologic diagnosis before the brush biopsy. Pathologic slides from previous tissue biopsies were evaluated retrospectively both for IHC p16 and HPV-18 and for low-risk genotypes HPV-6 and HPV-11 and compared with the results of the brush biopsy.^34^

### Statistical analysis

The distribution of patient characteristics, including demographics, social history, clinical oral condition, and HPV status, was tabulated using statistical software (JMP Pro 14; SAS Institute). Descriptive statistics, including median and range for continuous variables, as well as frequency for categorical information, were used to summarize the data.

The prevalence of HPV infections (any HPV, low-risk and high-risk types) was evaluated by sex, tobacco use, alcohol consumption, and clinical diagnosis; significant heterogeneity was assessed with a χ^2^ test. All *P* values were considered to be statistically significant at *P* < .05.

## Results

### Sociodemographic information

A total of 106 patients were screened from March 2016 through September 2017. Seven patients were excluded because of oropharyngeal lesions. Ninety-nine patients were included in this study. The median age was 57 years (range, 48-67 years), and 53 (53.5%) patients were females. When tobacco and alcohol consumption were considered, 50 (50.5%) of patients never smoked tobacco, and 59 (59.6%) had never had alcoholic beverages in their lifetimes (Table 1).

**Table 1:** Sociodemographic information of the patients.

Variables	Total (N = 99)	HPV∗ rtPCR† (n = 15)	*P* Value	rtPCR High Risk HPV (n = 10)	*P* Value	rtPCR Low Risk HPV (n = 6)	*P* Value	P16INK4a-Immunohistochemistry (n = 7)
Age, y, median (range)	57 (48-67)	58 (48-67)	.1576	59 (52-67)	.2470	56 (48-61)	.1576	61 (54-67)
Sex, no. (%)								
Female	53 (53.5)	5 (33.3)	.0886	3 (30.0)	.5334	2 (33.3)	.0886	2 (28.6)
Male	46 (46.5)	10 (66.6)		7 (70.0)		4 (66.6)		5 (71.4)
Tobacco use, no. (%)								
Never	50 (50.5)	4 (26.7)	.9652‡	3 (30.0)	.1660‡	1 (16.7)	.1687‡	3 (42.8)
Current	26 (26.3)	7 (46.6)		3 (30.0)		3 (50.0)		1 (14.4)
Former	23 (23.2)	4 (26.7)		4 (40.0)		2 (33.3)		3 (42.8)
Pack/years, median (range)	20 (1-84)	21 (10-80)		25 (10-80)		16 (10-60)		17 (4-40)
Alcohol consumption, no. (%)								
Never	59 (59.6)	7 (46.6)	.1921‡	5 (50.0)	.2598‡	2 (33.3)	.1775‡	3 (42.8)
Former	5 (5.1)	0 (0.0)		0 (0.0)		0 (0.0)		0 (0.0)
Current	35 (35.3)	8 (53.4)		5 (50.0)		3 (50.0)		4 (57.2)
Type of alcohol, no. (%)								
Beer or wine	37 (37.4)	8 (53.4)		5 (50.0)		3 (50.0)		4 (57.2)
Spirits	3 (3.1)	0 (0.0)		0 (0.0)		0 (0.0)		0 (0.0)

∗ HPV: Human papillomavirus.

† rtPCR: Real-time polymerase chain reaction.

‡ *P* value was calculated for the current or former vs never.

### Oral diseases: Clinical characteristics

OL was the most common oral lesion (40 patients [40.4%]), with 16 (40.0%) patients having localized homogeneous features, 14 (35.0%) having a diagnosis of proliferative leukoplakia, and 10 (25.0%) having OL with localized nonhomogeneous features. This was followed by conventional reticular oral lichen planus (OLP) in 21 patients (21.2%), OSCC in 17 (17.2%), and oral squamous papilloma in 10 (10.1%).

When the histopathologic diagnosis was considered, most of the 40 OLs (23 [80.0%]) were hyperkeratosis nonreactive [HkNR], 6 (15.0%) showed mild oral epithelial dysplasia, and 2 (5.0%) were invasive OSCC. Of the 99 patients, 19 had a histopathologic diagnosis of moderately differentiated OSCCs (19.2%).^32^ All 21 clinically diagnosed cases of OLP lesions (21.2%) were histologically confirmed, and among all 21 benign lesions, 11 (52.4%) were oral fibroma, 8 (38.1%) were squamous papilloma, and 2 (9.5%) were pyogenic granuloma. The most involved oral cavity site was the tongue in 42 patients (42.4%), with 17 (40.5%) showing a lesion of the tongue dorsum, 16 (38.1%) of the lateral aspect of the tongue, and 9 (21.4%) of the ventral surface. This was followed by the buccal mucosa (BM) in 40 patients (40.3%) and attached gingiva in 30 (30.2%) (Table 2).

**Table 2:** Clinical, histopathologic characteristics, and HPV∗ infection in oral mucosal lesions.†

Variables	Total, No. (%) (N = 99)	Sex, No. (%)		rtPCR‡ HPV, No. (%) (n = 15)§	*P* Value	rtPCR High Risk HPV, No. (%) (n = 10)¶	*P* Value	rtPCR Low Risk HPV, No. (%) (n = 6)¶	*P* Value	P16INK4a-Immunohistochemistry, No. (%) (n = 7)
		Male (n = 46)	Female (n = 53)							
Clinical diagnosis										
Oral leukoplakia	40 (40.4)	21 (45.6)	19 (35.8)	7 (46.6)	.1962	3 (30.0)	.0500	4 (66.6)	.1962	4 (57.1)
Localized	26 (65.0)	17 (80.9)	9 (47.4)	5 (71.4)		2 (66.6)		3 (75.0)		3 (75.0)
Proliferative	14 (35.0)	4 (19.1)	10 (52.6)	2 (28.6)		1 (33.3)		1 (25.0)		1 (25.0)
Oral lichen planus	21 (21.2)	9 (19.6)	12 (22.6)	5 (33.3)	.4984	4 (40.0)	.9908	1 (16.7)	.4984	1 (14.3)
Oral squamous cell carcinoma	17 (17.2)	8 (17.4)	9 (17.1)	0 (0.0)		0 (0.0)		0 (0.0)		1 (14.3)
Benign lesions	21 (21.2)	8 (17.4)	13 (24.5)	4 (26.6)	.8222	3 (30.0)	.2200	1 (16.7)	.8222	1 (14.3)
Oral fibroma	11 (52.4)	4 (50.0)	7 (53.8)	1 (25.0)		1 (33.3)		0 (0.0)		0 (0.0)
Squamous papilloma	8 (38.1)	3 (37.5)	5 (38.5)	1 (25.0)		1 (33.3)		0 (0.0)		1 (100.0)
Pyogenic granuloma	2 (9.5)	1 (12.5)	1 (7.7)	2 (50.0)		1 (33.3)		1 (100.0)		0 (0.0)
Anatomic site affected#										
Tongue	42 (42.4)	23 (50.0)	19 (35.8)	6 (40.0)		4 (40.0)		2 (33.3)		4 (57.1)
Dorsum	17 (40.5)	7 (30.4)	14 (73.7)	3 (50.0)		2 (50.0)		1 (50.0)		2 (50.0)
Lateral surface	16 (38.1)	11 (47.8)	3 (15.8)	2 (33.3)		1 (25.0)		1 (50.0)		1 (25.0)
Ventral surface	9 (21.4)	5 (21.7)	2 (10.5)	1 (16.7)		1 (25.0)		0 (0.0)		1 (25.0)
Buccal mucosa	40 (40.3)	17 (36.9)	23 (43.4)	7 (46.6)		4 (40.0)		3 (50.0)		1 (14.3)
Gingiva	30 (30.2)	13 (28.3)	17 (32.1)	5 (33.3)		4 (40.0)		1 (16.7)		3 (42.8)
Hard palate	9 (9.1)	3 (6.5)	6 (11.3)	3 (20.0)		2 (20.0)		1 (16.7)		0 (0.0)
Retromolar trigone	3 (3.0)	2 (4.3)	1 (1.9)	0 (0.0)		0 (0.0)		0 (0.0)		0 (0.0)
Lip mucosa	3 (3.0)	2 (4.3)	1 (1.9)	0 (0.0)		0 (0.0)		0 (0.0)		0 (0.0)
Floor of the mouth	1 (1.0)	0 (0.0)	1 (1.9)	0 (0.0)		0 (0.0)		0 (0.0)		0 (0.0)
Histopathologic diagnosis										
Hyperkeratosis nonreactive	32 (32.3)	17 (36.9)	15 (28.3)	5 (33.3)		2 (20.0)		3 (50.0)		0 (0.0)
Lichenoid mucositis	21 (21.2)	9 (19.6)	12 (22.6)	5 (33.3)		4 (40.0)		1 (16.7)		1 (14.3)
Invasive squamous cell carcinoma	19 (19.2)	10 (21.7)	9 (17.0)	0 (0.0)		0 (0.0)		0 (0.0)		3 (42.8)
Squamous papilloma	10 (10.1)	5 (10.9)	5 (9.4)	1 (6.6)		1 (10.0)		0 (0.0)		0 (0.0)
Fibroma	9 (9.1)	2 (4.3)	7 (13.2)	1 (6.6)		1 (10.0)		0 (0.0)		1 (14.3)
Mild epithelial dysplasia	6 (6.1)	2 (4.3)	4 (7.6)	2 (13.3)		1 (10.0)		1 (16.7)		2 (28.6)
Pyogenic granuloma	2 (2.0)	1 (2.3)	1 (1.9)	2 (13.3)		1 (10.0)		1 (16.7)		0 (0.0)

∗ HPV: Human papillomavirus.

† The total number of cases within each column corresponds to various clinical or histopathologic categories, including oral leukoplakia, oral lichen planus, tongue, buccal mucosa, hyperkeratosis. The subcategories (eg, homogeneous or nonhomogeneous leukoplakia or ventral or lateral tongue) display percentages that pertain to their respective category rather than the overall total.

‡ rtPCR: Real-time polymerase chain reaction.

§ Numbers add to 16 as 1 patient tested positive for 2 HPV genotypes.

¶ Leukoplakia: 2 (HPV-16), 1 (HPV-18), 1 (HPV-6), 1 (HPV-11), 1 (HPV-70); oral lichen planus: 3 (HPV-16), 1 (HPV-58), 1 (HPV-6); benign lesions: 2 (HPV-16), 1 (HPV-18), 1 (HPV-6); tongue: 3 (HPV-16), 1 (HPV-18), 1 (HPV-6), 1 (HPV-70); buccal mucosa: 3 (HPV-16), 1 (HPV-18), 2 (HPV-6), 1 (HPV-11); gingiva: 4 (HPV-16), 1 (HPV-6); hard palate: 1 (HPV-16), 1 (HPV-58), 1 (HPV-11); Hyperkeratosis nonreactive: 1 (HPV-16), 1 (HPV-18), 1 (HPV-6), 1 (HPV-11), 1 (HPV-70); lichenoid mucositis: 3 (HPV-16); 1 (HPV-58), 1 (HPV-6); mild epithelial dysplasia: 1 (HPV-16); squamous papilloma: 1 (HPV-18); fibroma: 1 (HPV-16); pyogenic granuloma: 1 (HPV-16), 1 (HPV-6); *P* value was calculated for HPV infection and clinical diagnosis.

# Numbers may add to more than 40 as several patients had multisite lesions.

### HPV Detection by rtPCR and p16 Detection by IHC

A total of 15 of 99 (15.2%) patients (10/15 [66.6%] males) had detectable HPV DNA, with most of the patients (10/15 [66.6%]) being positive for high-risk HPV genotypes: HPV-16 (7/10 [70.0%]), HPV-18 (2/10 [20.0%]), and HPV-58 (1/10 [10.0%]). The remaining patients (6/15 [40.0%]) were positive for low-risk HPV genotypes: HPV-6 (4/6 [66.6%]), HPV-12 (1/6 [16.6%]), and HPV-70 (1/6 [16.6%]) (sTable; available at the end of this article). One patient tested positive for both HPV-16 and HPV-6. There were no statistically significant differences in HPV positivity when sex, tobacco use, and alcohol consumption were considered.

Patients with OL showed the highest rate of HPV infection (7 [46.6%]; *P* = .1962), followed by patients with OLP (5 [33.3%]; *P* = .4984) and patients with benign lesions (4; [26.6%]; *P* = .8222). No patients with OSCC tested positive for HPV DNA via rtPCR. When the HPV genotypes were considered, patients with high-risk HPV genotypes (HPV-10, HPV-16, HPV-18, and HPV-58, respectively) had a diagnosis of OLP in 4 of 10 (40.0%) cases (HPV-16, 3; HPV-58, 1; *P* = .9908), 3 of 10 (30%) patients were affected by OL (HPV-16, 2; HPV-18, 1; *P* = .0500), and 3 of 10 had an oral benign lesion (30.0%; HPV-16, 2; HPV-18, 1; *P* = .2200). Low-risk HPV infections were detected in 6 of 15 patients overall, most of whom had a clinical diagnosis of OL (4/6 [66.6%]; HPV-6, 2; HPV-12, 1; HPV-70, 1; *P* = .1962). Similarly, when the histopathologic data were considered, the highest rate of HPV infection was found in patients having HkNR with no dysplasia (5 [33.3%]; HPV-16, 1; HPV-18, 1; HPV-6, 1; HPV-12, 1; HPV-70, 1), and lichenoid mucositis (5 [33.3%]; HPV-16, 3, HPV-58, 1; HPV-6, 1), followed by patients with oral epithelial dysplasia (2 [13.3%]; HPV-16, 1; HPV-6, 1). None of the patients with a histologically confirmed OSCC resulted positive for HPV DNA detected by rtPCR.

Patients with lesions on the BM had the highest rate of HPV infection (7/15 [46.6%]; HPV-16, 3; HPV-18, 1; HPV-6, 2; HPV-11, 1); this was followed by patients with lesions of the tongue (6/15 [40.0%]; HPV-16, 3; HPV-18, 1; HPV-6, 2; HPV-11, 1), and gingiva (5/15 [33.3%]; HPV-16, 4; HPV-6, 1) (Table 2).

When the p16 expression was investigated, a total of 7 of 99 (7.1%) patients showed a positive cytoplasmic expression with different grades (sTable). However, only 1 patient (with a clinical diagnosis of localized homogeneous leukoplakia of the ventrolateral tongue and exhibiting mild epithelial dysplasia) showed a truly positive p16 expression (p16, 75% of cytoplasmic expression).^35^ This patient also tested positive for rtPCR HPV-16.

## Discussion

With the increasing burden of HPV-related OPC, we sought to understand better the prevalence of HPV infections in patients affected by oral diseases.^36^^,^^37^ In particular, we evaluated the HPV status (rtPCR and IHC) of 99 patients with OPMDs, oral benign lesions, and OSCC. Overall, 15 of the 99 (15.2%) patients had HPV infection, with most them being positive for high-risk HPV genotypes (10 [66.6%]) and 5 of 15 (33.4%) being positive for low-risk HPV genotypes. Only 1 patient was p16 positive, showing a cytoplasmic protein expression of 75%.

A 2023 large study (n = 3,196) conducted in dental offices in the United States showed that the prevalence of oral HPV was 6.6%.^38^ Our research yielded a notably higher HPV prevalence of 15.2% among patients with oral lesions. This disparity may suggest a possible association between oral HPV infections and specific oral conditions, including some OLs and OLP. Similar findings were reported in a 2020 meta-analysis, in which more than 29% of OLP cases were positive for high-risk HPV genotypes, showing a 5-times higher risk of HPV infection than control patients (odds ratio, 4.91; 95% CI, 2.76 to 8.72); and 31.6% of HPV-positive OL cases had a 2.5-times higher risk of HPV infection than control patients (odds ratio, 2.51; 95% CI, 1.55 to 4.07).^39^ When the association between high-risk HPV infection and subsets OLP and OL was assessed, patients with erosive atrophic OLP were found to have a 5.3-times higher risk than reticular OLP cases and control patients. None of our patients had a clinical diagnosis of atrophic or erosive lichen planus, but the oral HPV prevalence was high (33.3%). In addition, patients with nonhomogeneous OL were found to have a 3.3 times higher risk of high-risk HPV infection than control patients.^39^ The higher HPV positivity in patients with oral lesions than in patients with no oral lesions may potentially be influenced by distinct sexual behaviors. However, we did not gather any sexual history data and could not verify this hypothesis. Another plausible explanation could be related to potential genetic susceptibility among hosts to HPV infections. However, we did not conduct any gene association studies to identify individual predisposition to oral HPV infections, and this hypothesis should be further investigated through future research.^40^ A 2015 case-control study evaluated the prevalence of HPV infection among 55 patients with histologically confirmed OLP. Overall, 11 of 40 (27.5%) cases were HPV positive, with most of them (8/11) being positive for high-risk HPV (HPV-16 and HPV-18).^41^ Our study showed similar results, with 33.3% of our OLP patients testing positive for HPV-16, HPV-58, and HPV-6. It is challenging to explain why a condition driven by a T-cell mediated immunologic reaction shows such rates of HPV infection; a plausible explanation could be that the degeneration of the epithelial basal cell layer might ease the viral infection or could be the consequence of localized immunosuppression secondary to corticosteroid or immunomodulators treatment.

Another study investigated the rates of HPV-16 infection in 83 patients with OPMDs and 106 patients with OSCC.^25^ Overall, the HPV-16 infection rates were particularly low but higher in patients with OSCC (7.5%) than in patients with OPMDs (3.6%), although not statistically significant (*P* = .2600). The authors also evaluated the rates of p16INK4a positivity, but only 61% of OSCC cases (65/106). A total of 19 (29.0%) OSCCs tested positive for p16INK4a; however, no cytoplasmic expression of the protein was determined.^35^ Therefore, it was not possible to determine the true validity of the results. In addition, OPMD was evaluated as a whole, and no distinction was made between the different clinical (ie, homogeneous vs nonhomogeneous leukoplakia vs OLP) and histopathologic diagnoses (ie, HkNR vs oral epithelial dysplasia vs OLP).

In 2017, Lerman et al^42^ conducted a prospective study and found a strong p16 positivity and high prevalence of HPV-16 in patients with a clinical diagnosis of OL (55) and a histopathologic diagnosis of oral dysplasia. All the patients showed a strong p16 positivity, with HPV-16 found in 20 of 22 (91%) patients. When the anatomic site of the HPV-positive patients was considered, the floor of the mouth was the most common area (77%) involved, followed by BM (14%) and gingiva (8%). HPV-16 was the most common genotype detected (91%), followed by HPV-33 and HPV-58. Among these 55 patients with HPV-positive oral epithelial dysplasia, 8 of 53 (15%) developed an HPV-positive OSCC. In our work, OL patients had the highest rate of oral HPV infection (40.0%), one-half of which were high-risk types (HPV-16, HPV-18), and 1 patient was positivite (> 75%) for the p16 staining. Even patients with OL with a histopathologic diagnosis of HkNR (71.4%) were HPV positive, suggesting that HPV positivity may be unrelated to the histopathologic diagnosis rather than be associated with the clinical diagnosis of leukoplakia only.

The anatomic distribution of HPV infection has been documented in broader regions (such as the oral cavity, oropharynx, and genital tract). However, there is a relative dearth of information regarding specific, localized areas within the oral cavity, such as the BM, tongue, and hard palate. Most studies tend to focus on mapping the anatomic distribution of clinical lesions rather than delving into the distribution of HPV infections within these specific oral sites.^43^ Nevertheless, Mattila et al,^44^ in their prospective study, evaluated the prevalence of HPV infection in 82 patients with OLP; overall, 84.1% of patients were found to be HPV-positive, with the BM being the most common anatomic site (88.3%), followed by the tongue (76.9%) and marginal gingiva (71.4%). Another prospective study conducted in 46 South American women with HPV-positive cervical intraepithelial neoplasia without oral cavity lesions showed a high prevalence of oral HPV infection of the hard palatal mucosa (38 [87%]; HPV 16, 7 [16%]) and of the BM (37 [86%]; HPV 16, 10 [23%]).^45^ Similarly, a 2016 study reported a high detection of HR HPV loads in patients with OL (66.7%) and OLP (25%), most of which were detected on the BM (70%), hard palatal mucosa (37.5%), and soft palate (42.9%).^31^ Consistent with the studies mentioned above, we found that OL and OLP were the most common clinical diagnoses among HPV-positive patients. In terms of anatomic distribution, the BM proved to be the most prevalent site of HPV infection at 46.6%, followed by the tongue at 40.0%, and the gingiva at 33.3%. The oral cavity can serve as potential reservoirs for HPV, with sites such as inflamed gingival pockets and the ductal epithelium of the salivary glands being implicated.^46^, ^47^, ^48^ This could provide an explanation for the observed higher rates of oral HPV in the BM (which is in close proximity to Stensen duct). In addition, it is worth considering that variations in sampling techniques by different operators may have contributed to these differences in HPV detection rates.

Our study had few limitations. First, we included only a small cohort of patients, making it difficult to generalize the results to the whole population. Second, we included patients with a history of tobacco smoking, a known risk factor for oral HPV infections. Third, we did not have a control group without oral lesions. Fourth, we did not consider the HPV vaccination status of the patients included in the study. It may be the patients with a history of immunization had lower rates of HPV infection.

Moreover, of all HPV types, those that cause oral squamous papilloma comprise more than 40 genotypes, with HPV-16 being the most frequently detected in potentially malignant and malignant diseases. Hence, it is not surprising that the highly sensitive but genotype-specific rtPCR used in our study, which has a limited target of HPV genotypes, tested negative in most squamous papillomas that are benign proliferative lesions. Finally, for those lesions that affected multiple sites of the oral cavity (such as oral proliferative leukoplakia and OLP), we could not determine the exact anatomic location of the viral settlement as the brush biopsy was performed at different oral mucosal sites. Nevertheless, the diagnostic tests were performed and evaluated by trained investigators, which may have improved the overall study outcomes.

## Conclusions

Although the association of HPV high-risk type infection for OPC is well established, the relationship between HPV infection and oral diseases remains poorly understood. In our study, we assessed the prevalence of HPV infections in a cohort of patients with oral benign pathology, OPMD, and OSCC. We found that the highest rate of HPV infection was in male patients and people affected by OL or OLP. When the anatomic site within the oral cavity was considered, HPV infection was more prevalent in lesions of the BM. Larger multicenter prospective studies with follow-up HPV tests are necessary to understand better the role of HPV infections in the pathogenesis of oral diseases. In particular, progression-type studies are essential to improve knowledge of the relatively high prevalence of HPV and establish a more comprehensive understanding of the role of HPV in oral diseases.

## Disclosure

None of the authors reported any disclosures.

**sTable:** Characteristics of HPV∗-positive patients.

Sex	Age, Y	Oral Lesion	Anatomic Location	HPV Type	HPV Copies	E2 HPV-16 Copies	E2-E6	P16INK4a-Immunohistochemistry, %†
Male	44	Homogeneous leukoplakia; mild dysplasia	Ventral tongue	HPV-16	25269.64	13233.2	1.57035581	75
Male	60	Oral squamous cell carcinoma	Gingiva	NA†	NA	NA	NA	60
Male	68	Proliferative leukoplakia	Gingiva	NA	NA	NA	NA	25
Male	48	Nonhomogeneous leukoplakia	Lateral tongue	HPV-16	2560.48	15322.58	5.98426076	15
Female	80	Oral lichen planus	Gingiva or buccal mucosa	HPV-16	4950.82	8108.93	1.63789635	10
Male	60	Nonhomogeneous leukoplakia	Tongue dorsum	HPV-16	8426.88	13233.2	1.57035581	5
Male	61	Oral fibroma	Tongue dorsum	HPV-16	5324.13	134.28	0.025	3
Male	38	Pyogenic granuloma	Gingiva	HPV-16	1350.95	2.30947	0.03171102	0
Male	80	Oral lichen planus	Gingiva	HPV-16	7548.66	12455.4	1.7680249	0
Female	56	Squamous papilloma	Tongue dorsum	HPV-18	32000000	NA	NA	0
Female	54	Homogeneous leukoplakia	Buccal mucosa	HPV-18	32502.7	NA	NA	0
Male	78	Oral lichen planus	Hard palate	HPV-58	4780.95	NA	NA	0
Male	47	Homogeneous leukoplakia	Tongue dorsum	HPV-70	35135	NA	NA	0
Male	63	Homogeneous leukoplakia	Lateral tongue	HPV-6	1321212	NA	NA	0
Female	66	Homogeneous leukoplakia; mild dysplasia	Buccal mucosa	HPV-6	1321212	NA	NA	0
Male	56	Oral lichen planus	Buccal mucosa	HPV-6	163.09	NA	NA	0
Male	38	Pyogenic granuloma	Gingiva	HPV-6	3495.93	NA	NA	0
Female	52	Proliferative leukoplakia	Gingiva	HPV-12	470.2	NA	NA	0

∗ HPV: Human papillomavirus.

† NA: Not applicable.
